# Fully Biobased Thermoset Adhesive Precursor from Itaconic Acid and Propylene Glycol

**DOI:** 10.1021/acsomega.5c10583

**Published:** 2025-12-17

**Authors:** Vojtěch Jašek, Eliška Kameníková, Kamil Novotný, Radek Přikryl, Silvestr Figalla

**Affiliations:** † Institute of Materials Chemistry, Faculty of Chemistry, 48274 Brno University of Technology, 612 00 Brno, Czech Republic; ‡ Materials Research Centre, Faculty of Chemistry, Brno University of Technology, 612 00 Brno, Czech Republic; § Chair of Materials Science and Testing of Polymers, 27268 Montanuniversität Leoben, Franz Josef-Straße 18, 8700 Leoben, Austria

## Abstract

Photocurable adhesives serve numerous practical applications, such as medical devices, electronics, sealing in LCDs, or glass bonding. This work focuses on a scalable and sustainable engineering of a new reactive precursor for photocurable adhesives, dipropylene glycol itaconate (DPG-IA). Synthesis involved Fischer esterification, with more than 98% conversion measured by convenient and affordable volumetric analyses (acid number and hydroxyl value). The product’s structural verification was provided by ^1^H Nuclear Magnetic Resonance (NMR) and FT-IR analysis, confirming a highly pure yield (more than 99%). Particularly, ^1^H NMR and FT-IR uncovered the presence of unsaturated double bonds, which can by radically polymerized and from cured molecular structure. The rheological profile and curing reactivity were investigated since they are crucial for photocurable adhesives. The apparent viscosity reached 1050 mPa·s at 25 °C, and the calculated flow activation energy reached 70.1 kJ/mol. DPG-IA’s reactivity was studied by Differential Scanning Calorimetry (DSC), uncovering the polymerization activation energy reaching 115 kJ/mol. Eventually, the adhesion strength, achieved by the cured DPG-IA on the acryl, wood, and glass adherents, was investigated. The measured adhesion strength for the acryl-wood adhesion reached 53.6 ± 1.5 kPa, and for the acryl-glass system it reached 83.4 ± 4.4 kPa.

## Graphical abstract

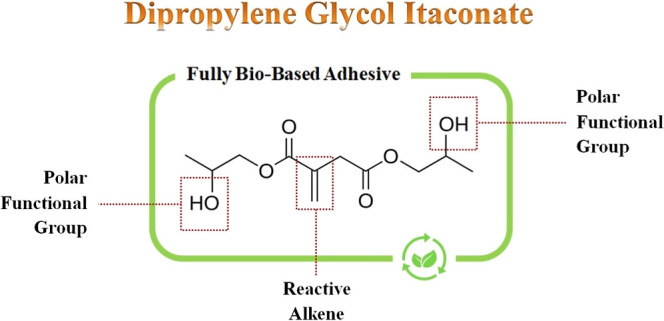

## 1 Introduction

Biobased and renewable materials attract enormous attention nowadays due to the legislative changes heading toward more sustainable manufacturing and industry. Several companies (particularly in the European Union) will have to incorporate more recycled or renewable content into their products to meet government requirements. Many industrial and application fields are affected by these new obligations, such as the automotive, composite, aerospace, and furniture industries.^1,2^ Adhesives are widely used for many particular purposes in many industrial fields; therefore, the specific research and inventions are investigated to incorporate more recycled or renewable content into the adhesive precursors.^3^ Currently, adhesives mainly comprise entirely fossil-based reactants, leading to the eventual products based on acrylates, cyanoacrylates, silicones, epoxides, or polyurethanes.^4^ Adhesives, such as cyanoacrylates or silicones, represent monocomponent systems requiring only application and curing time for their optimal function.^5,6^ On the other hand, epoxides demand specific reactive additives (amines) to form an appropriate functional adhesive.^7^ Polyurethane systems are commonly composed of a polyol and an isocyanate component.^8^ Regarding cyanoacrylic adhesives, any potential incorporation of the recycled or renewable content is problematic due to the chemical nature of the system. Cyanoacrylates are usually synthesized via the reaction of the alkyl ester of cyanoacetate and formaldehyde.^9^ This production can hardly be connected to a more sustainable approach. On the other hand, there are several biobased epoxides such as limonene epoxide, lignin epoxy derivatives, or modified vegetable oils or fatty acid esters.^10,11^ Polyurethanes may comprise polyols from recycled or renewable sources such as appropriate carbohydrates or glyceride derivatives.^11^ Additionally, there are many investigated systems based on the nonisocyanate polyurethanes manufactured from carbonated biobased epoxides.^11^

Acrylates make up a specific group of adhesive-forming compounds. These molecules can be polymerized and serve as pressure-sensitive adhesives, which require a particular solvent for dissolution, leading to the applicability on the adherent, separation of the assisting solvent, and eventually working as an adhesive.^12^ The radical curability with suitable photoinitiators is an alternative application approach. The radically polymerizable precursor is homogenized with a photoinitiator, and once this system is exposed to a specific irradiation, it forms a thermoset polymeric structure working as a connecting binder.^13^ Several modified fatty acids or glycerides are studied in the available literature to fulfill this purpose. Unsaturated fatty acids in glycerides’ structure comprise double bonds, which are modifiable to epoxy, acrylic, methacrylic, or other functional groups.^14^ Typically, the presence of polar functional groups such as hydroxyls, esters, ethers, or amides is critical for the best potential adhesive performance.^14,15^ Usually, the glyceride derivatives possess a strong hydrophobic character due to their long hydrocarbon chains, lacking any polar functional groups.^14^ Itaconic acid (IA) derivatives are a specific group of compounds connecting the entirely biobased character with the opportunity for radical polymerization. IA is produced through microbial fermentation (*Aspergillus terreus* is commonly used for the fermentation).^16^ At the same time, the unsaturated double bond within its structure ensures its reactivity when triggered with an appropriate initiator.^17^

Next to the itaconic-based precursors and adhesive-forming compounds, the sustainable materials come from other sources, such as starch or lignocellulose. Oktay et al.^25^ investigated a thermally cured adhesive from cornstarch, Mimosa tannin, sugar, and citric acid as a sustainable alternative to fossil-based systems for binding applications. Their product was composed entirely of renewable sources capable of forming a cross-linked structure suitable for adhesives. Moreover, the authors proved that their suggested synthesis fulfills the permissible formaldehyde content, which may occur during the high-temperature production route. Another investigation studied the combination of natural red pine tannin (RT) in combination with hexamethylenetetramine (HMTA), resulting in the tannin-based resin formulation. The observations confirmed that the suggested system formed a cross-linked network appropriate for adhesive purposes.^26^

In this study, we present a fully biobased itaconic acid derivative, involving propylene glycol from renewable sources. The synthesized dipropylene glycol itaconate (DPG-IA) possesses optimal viscosity, a number of polar functional groups, and curability, ensuring its potential in the adhesive industry. We also synthesized a well-known methacrylated vegetable oil as a reference reactive precursor to our proposed innovative DPG-IA.

## 2 Experimental Section

### 2.1 Materials

Itaconic acid (99%), ethyl acetate (99%), sulfuric acid (96%), Luperox DI for the Differential Scanning Calorimetry (DSC) measurements (*tert*-butyl Peroxide, 98%), and BAPO (photoinitiator, phenylbis­(2,4,6-trimethylbenzoyl)­phosphine oxide, 98%) were purchased from Sigma-Aldrich. The biobased propylene glycol (99.7%) produced from the plant-based glycerol was obtained from Orlen (Poland).

### 2.2 Dipropylene Glycol Itaconate (DPG-IA) Synthesis

Itaconic acid (1 mol, 130 g) and propylene glycol (4 mol, 304 g) were loaded into a three-neck bottom flask and preheated to 100 °C. After the homogenization, sulfuric acid (0.01 mol, 0.98 g) was added to the mixture. The reaction was performed for 4 h. During the reaction, the acid number was measured, and the reaction water was collected. The water distillation involved reduced pressure (60 Torr). After the reaction, excess propylene glycol was separated by liquid–liquid extraction (LLE). The postreaction solution was diluted with ethyl acetate (1:1 volume ratio). The diluted mixture was washed with water (twice, 1:1 volume ratio to the diluted postreaction mixture). After the LLE, the used ethyl acetate was distilled to obtain the purified DPG-IA. The obtained dipropylene glycol itaconate was structurally verified by FT-IR, the acid number, and the hydroxyl value.

### 2.3 Characterization Methods

#### 2.3.1 Nuclear Magnetic Resonance

Nuclear magnetic resonance (NMR) was applied to obtain ^1^H spectra to confirm the synthesized DPG-IA’s chemical structure. The measurements were conducted by a Bruker Avance III (Bruker, Billerica, MA, USA). The measuring frequency was 500 MHz for ^1^H NMR. The measurements were performed at 30 °C temperature using d-chloroform (CDCl_3_) as a solvent with tetramethylsilane (TMS) as an internal standard. The chemical shifts (δ) are expressed in parts per million (ppm) units, referenced by a solvent. Coupling constant (*J*) is expressed with frequency unit (Hz), with coupling expressed as s—singlet, d—doublet, t—triplet, q—quartet, p—quintet, and m—multiplet.

#### 2.3.2 Fourier-Transform Infrared Spectrometry

Fourier-transform infrared spectrometry (FT-IR) was used as a structure verification method. FT-IR spectrum served as one cross-analysis for isosorbide monomethacrylate structure verification and to describe the structural changes in MISD-containing commercial Polipol 3870 resins. The instrumentation was a Bruker Tensor 27 (Billerica, MA, USA), and the applied method was attenuated total reflectance using diamond as a dispersion component. The diode laser was an irradiation source. A Michelson interferometer was used to quantify the signal. Spectra comprised 32 total scans with a measurement resolution of 4 cm^–1^.

#### 2.3.3 Acid Number Determination

Acid number (A.N.) quantifies the number of acidic functional groups. The sample (0.1–0.3 g) is diluted in the appropriate solution (acetone), and the pH indicator (bromothymol blue) is added to the mixture. 0.1 M potassium hydroxide in methanol is used as a titration solution. The calculation is shown in eq 1:

1 $$A.N.=\frac{c_{KOH}\cdot V_{KOH}\cdot 56\;100}{m_{sample}}$$

where A.N. is the acid number (mg KOH/g), *c*_KOH_ is the molar concentration of the titration solution (mol/dm^3^), *V*_KOH_ is the volume of the titration solution (dm^3^), and *m*_sample_ is the weight of the measured sample (g).

The theoretical acid number calculated for the starting reactant, itaconic acid, followed eq 2:

2 $$A.N.\left(Theor.\right)=\frac{n_{COOH}\cdot 56\;100}{M_{r}}$$

where A.N. (Theor.) is the theoretical acid number (mg KOH/g), *n*_COOH_ is the theoretical number of acidic functional groups (−), and *M*_r_ stands for the theoretical molecular weight of the compound (g/mol).

#### 2.3.4 Hydroxyl Value Determination

Hydroxyl value (H.V.) is the quantity of hydroxyl functional groups occurring in a chemical structure. The principle of determination is the acetylation of vacant hydroxyl groups via acetic anhydride in the presence of pyridine as a catalyst. The sample (0.25–0.5 g) is mixed with 5 mL of 25% w/w solution of acetic anhydride in pyridine. The mixture is tempered at 100 °C for 1 h. The solution is mixed with 10 mL of water after the reaction to hydrolyze excess anhydride. The mixture is titrated with 1 M potassium hydroxide solution in water. Bromothymol blue is used as an indicator. The calculation of hydroxyl value is provided in eq 3:

3 $$H.V.=\frac{\left(V_{BLANK}-V_{KOH}\right){\cdot c}_{KOH}\cdot 56\;100}{m_{sample}}$$

where H.V. is the hydroxyl value (mg KOH/g), *c*_KOH_ is the molar concentration of the titration solution (mol/dm^3^), *V*_BLANK_ is the volume of the titration solution for blank (dm^3^), *V*_KOH_ is the volume of the titration solution for sample (dm^3^), and *m*_sample_ is the weight of the measured sample (g).

The theoretical hydroxyl value calculated for the starting reactant, itaconic acid, followed eq 4:

4 $$H.V.\left(Theor.\right)=\frac{n_{COOH}\cdot 56\;100}{M_{r}}$$

where H.V. (Theor.) is the theoretical hydroxyl value (mg KOH/g), *n*_OH_ is the theoretical number of hydroxyl functional groups (−), and *M*_r_ stands for the theoretical molecular weight of the compound (g/mol).

### 2.4 Differential Scanning Calorimetry for the Reactivity Study

DSC confirmed the curability of DPG-IA. The sample was mixed with Luperox DI (*tert*-Butyl peroxide, 1% (w/w) quantity to product). The solutions were transferred to aluminum pans (6–7 mg) and hermetically sealed. The instrument (DSC 2500 model from TA Instruments (New Castle, DE, USA)) was used for measurements. Four heating ramps were applied to each sample (10 to 200 °C) with ramps of 5, 10, 15, and 20 °C·min^–1^. We applied Kissinger’s reactivity theory introduced in eq 5:

5 $$\ln\left(\frac{\beta }{{T_{p}}^{2}}\right)=\ln\left(\frac{AR}{E}\right)-\frac{E}{R}\cdot \frac{1}{T_{p}}$$

where β is the heating rate (°C/min), *T*_p_ is the exothermic peak temperature (°C), *A* is the pre-exponential factor (−), *E* is the activation energy of the reaction (J/mol), and *R* is the gas constant (J/(mol·K)).

### 2.5 Rheological Investigation

DPG-IA’s rheological behavior was monitored by a TA Instruments rheometer AR-G2 rotational viscometer to describe its flow profile. The apparent viscosity dependency on the temperature was measured and rearranged to obtain Arrhenius parameters that are essential for the rheological description. The measurements used a Peltier platform and cone–plate geometry (40 mm with a 2° angle). The method was set as follows: shear rate 100 s^–1^ and temperature gradient 25–60 °C. The applied sample volume was 500 μL. The Arrhenian plot (1) is formulated as follows (eq 6):

6 $$\ln\;\eta =\frac{E_{\eta }}{R}\cdot \frac{1}{T}+\ln{\;\eta }_{\infty }$$

where the dependence of apparent viscosity ln (η) (−) on the reverse value of temperature 1/*T* (K^–1^) is constructed, we can obtain the flow activation energy *E*_η_ (J/mol) from the slope by multiplying it by the universal gas constant *R* (J/(mol·K)). Also, we can extract the infinite-temperature viscosity η_∞_ (Pa s) from the y-intercept by applying an exponential operation.

### 2.6 Adhesion Strength Test

The adhesion strength was determined according to SN EN 1465 (668510) in a Zwick/Roell 500 N (Ulm, Germany) testing machine under displacement control with a test speed of 1 mm/min. The adhesion area was 25 × 25 mm. The equation determining the adhesion strength (σ_Adhesion_) is formulated as follows (eq 7):

7 $$\sigma_{Adhesion}=\frac{F_{\mathrm{MAX}}}{A}$$

where σ_Adhesion_ is the adhesion strength (MPa), *F*_MAX_ represents the maximum force at adhesion break (N), and *A* stands for the adhesion area of the specimen (mm^2^).

## 3 Results and Discussion

### 3.1 Synthesis and Structural Characterization

The full biobased photocurable adhesives attract much attention due to the current requirements of the material industry segment. Currently, natural resources or recycled/upcycled entering substances are preferred for the manufacture due to the projected sustainable approach. Also, since itaconic acid and propylene glycol exhibit considerably lower environmental and health hazards compared to other substances used for the preparation of the photocurable precursors, the proposed produced dipropylene itaconate (DPG-IA) represents a safer alternative to the currently applied fossil-based acrylates or methacrylates. We prepared DPG-IA via Fischer esterification, which produces the reaction water as a secondary product. During this synthesis, the overall acidity of the system decreases due to the disappearance of the carboxyl groups reacting with the free hydroxyls, producing esters. The gravimetrical determination of the reaction water, together with the acidity value monitoring, is summarized in Figure 1. The obtained ^1^H NMR spectrum confirming the synthesized DPG-IA’s structure is also included in Figure 1.

**Figure 1:**
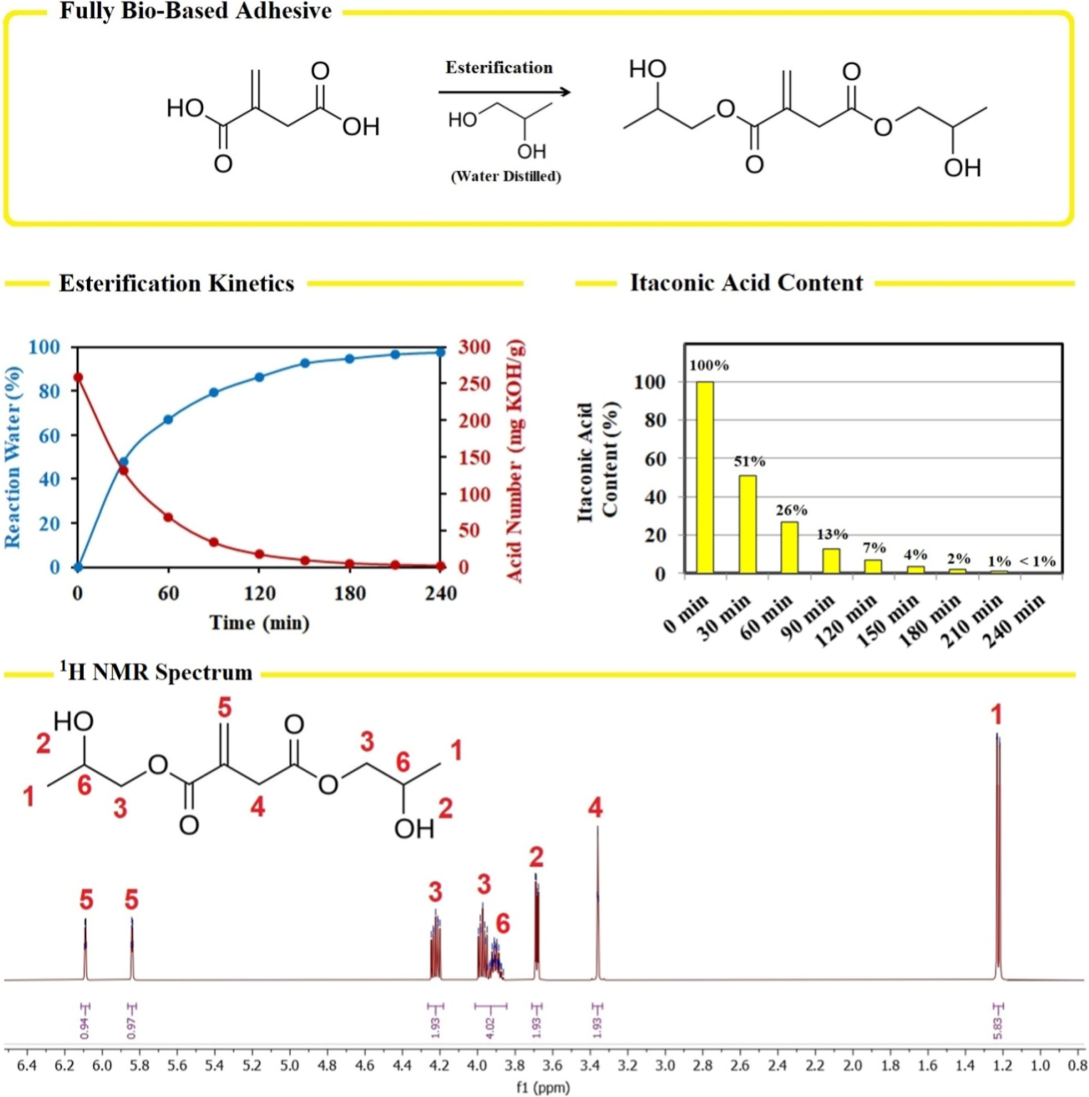
Reaction schemes for dipropylene glycol itaconate (DPG-IA, top). The investigated reaction kinetics composed of the measured acid numbers and the reaction water collection during the syntheses (middle). ^1^H NMR spectrum of the synthesized DPG-IA (bottom) (500 MHz, chloroform-*d*): δ 6.09 (dt, *J* = 2.3, 1.1 Hz, 1H), 5.84 (dt, *J* = 2.2, 1.0 Hz, 1H), 4.22 (ddd, *J* = 11.6, 6.3, 5.0 Hz, 2H), 4.01–3.84 (m, 4H), 3.68 (dd, *J* = 5.7, 2.4 Hz, 2H), 3.36 (d, *J* = 1.8 Hz, 2H), 1.22 (dd, *J* = 7.0, 1.0 Hz, 6H).

Figure 1 summarizes the reaction scheme, leading to dipropylene glycol itaconate (DPG-IA) through Fischer esterification. During this reaction, water is generated as a secondary product, which is continually distilled to progress the reaction effectively, following Le Chatelier’s principle. The reaction water yield is quantified in Figure 1 (middle left, blue graph), resulting in a 97.5% yield. A similar trend was uncovered in the volumetric analysis of the acid number, quantifying the itaconic acid content in the mixture. After the reaction time, the esterified IA reached a conversion of 99.2%. Both measurements confirmed the successful esterification, resulting in DPG-IA formation. The solventless Fischer esterification has not yet been studied in connection with itaconic acid derivatives. Typically, a Dean–Stark dehydration apparatus is used, which decreases the scalability and sustainability of the process due to the supporting solvent, usually increasing VOCs.^18^ Figure 1 (bottom) also contains ^1^H NMR analysis, confirming the chemical structure of the synthesized DPG-IA. The FI-IR method was used to verify the functional structure of the formed DPG-IA. The results comparing the entering itaconic acid with the formed diester are illustrated in Figure 2.

**Figure 2:**
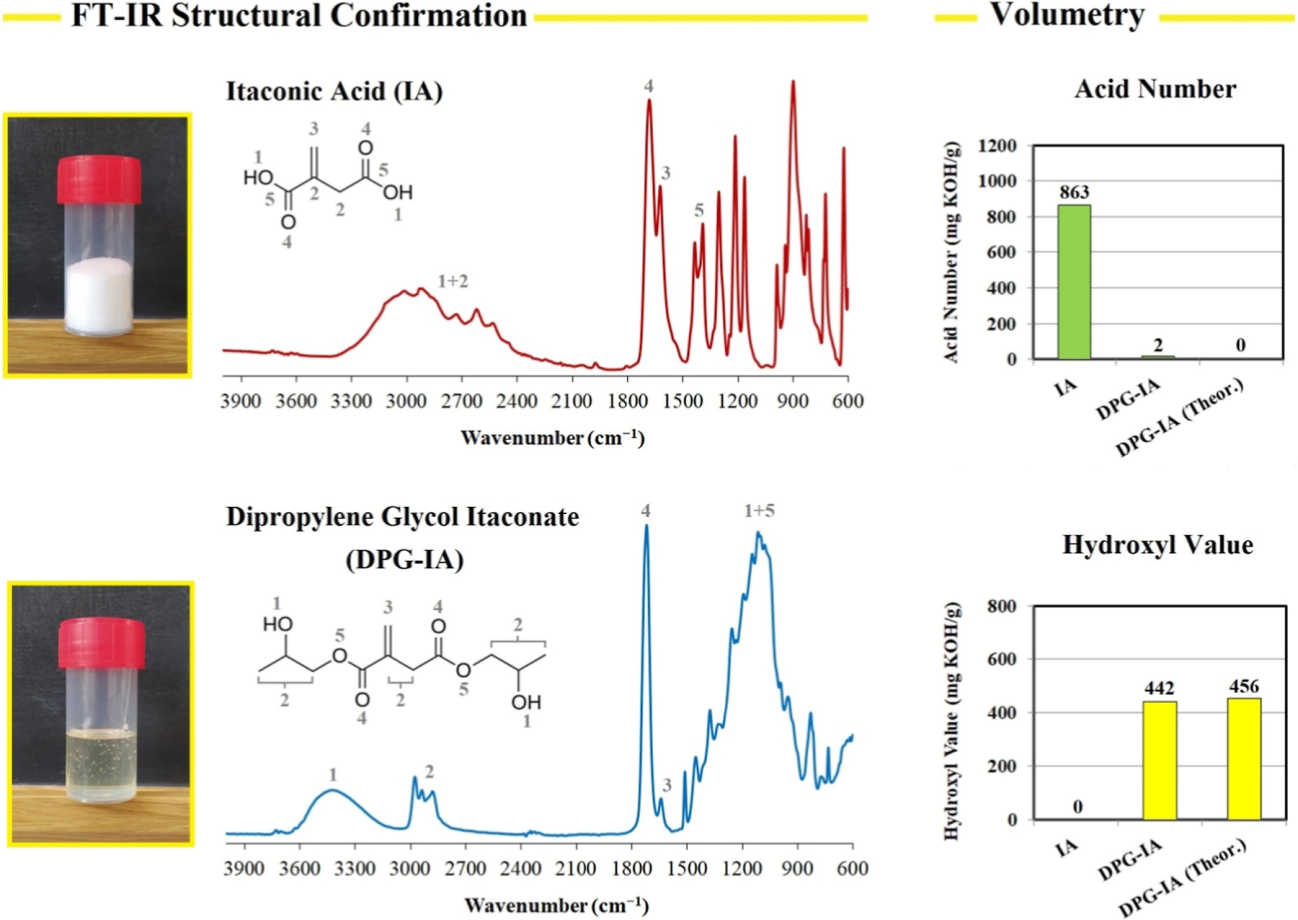
FT-IR analysis of the entering itaconic acid (IA) and the synthesized dipropylene glycol itaconate (DPG-IA). The acid number and hydroxyl value analyses verify DPG-IA’s structure by comparing the values with the theoretical.

The acquired FT-IR spectrum of itaconic acid uncovers the broad stretching signal of −OH bonded in carboxylic acid (3300–2500 cm^–1^) merged with the C–H stretching signal (3100–2840 cm^–1^). The C=O stretching signal confirming the carboxylic bonding (1700–1680 cm^–1^) differs from the signal in the DPG-IA spectrum (1730–1715 cm^–1^), which verifies the conversion of a carboxylic acid to an ester. The alkene stretching signal (1680–1660 cm^–1^) and the O–H bending signal (1440–1390 cm^–1^) also confirm the initial IA molecular structure. The formed DPG-IA contains a separate −OH stretching signal (3600–3200 cm^–1^), which differs compared to the IA spectrum. This hydroxyl signal change contributes to the structural confirmation together with the volumetric analyses. The measured hydroxyl value (442 mg KOH/g) corresponds with the theoretical value calculated for DPG-IA. Also, the acid number contributes to the structural confirmation since the acidity disappeared in the DPG-IA sample (measured 2 mg KOH/g). Besides the stretching carbonyl group switch from the carboxyl to ester, the DPG-IA spectrum contains the alkene stretching signal (1680–1660 cm^–1^). The C–O stretching signal (occurring in the ester and alcohol bond) also appears in the DPG-IA spectrum (1300–1000 cm^–1^). The combination of FT-IR and volumetric analyses verifies the successful and quantitative esterification of IA to DPG-IA.

### 3.2 Rheological Characterization and Reactivity

The investigated rheological profile of DPG-IA is illustrated in Figure 3 (top). We measured the apparent viscosity dependency on the shear rate, which uncovered that DPG-IA exhibits a Newtonian rheological behavior. The viscosity measured at 25 °C reached 1050 mPa·s and decreased with the rising temperature, as illustrated Figure 3 (top, right). According to the Arrhenius equation, we calculated the flow activation energy (*E*_η_) (reaching 70.1 kJ/mol). The measured rheological profile reflects the high intermolecular forces generated by free hydroxyl groups. This functional group forms hydrogen bonding and provides dipole–dipole (Keesom) interaction, which affects the flow profile enormously.^19^

**Figure 3:**
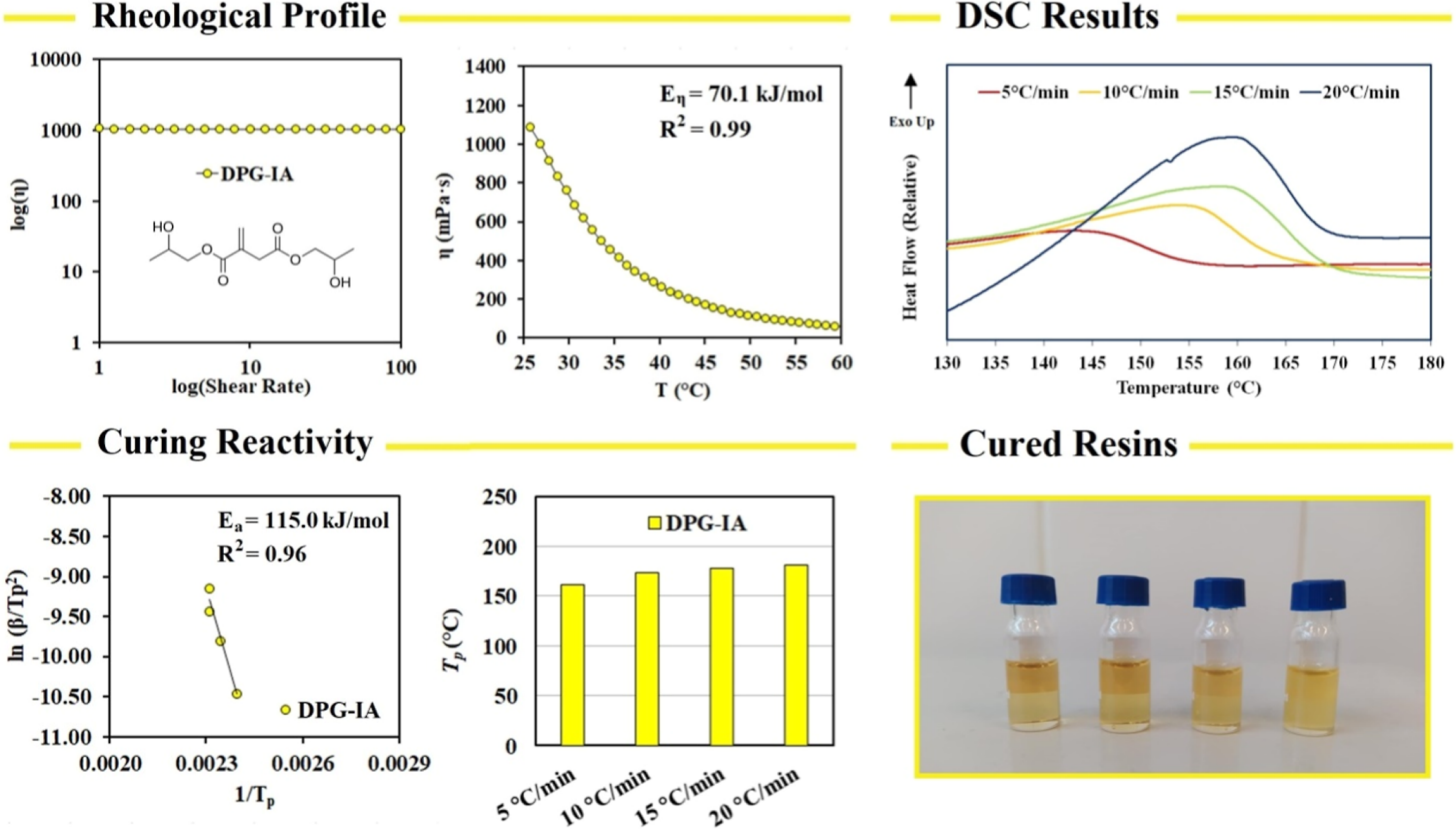
Rheological characterization and curing reactivity investigation of the synthesized dipropylene glycol itaconate (DPG-IA).

We also investigated DPG-IA’s curing reactivity before the adhesion performance study. The relative DSC graphs are shown in Figure 3 (top right). The increasing heating temperature ramp corresponds with the rising relative maximum peak temperature, as described in other papers investigating curing kinetics by DSC.^27,28^ This phenomenon is caused by the reaction progressing at a faster rate of temperature increase. The present thermal initiator requires less time to fully decompose, while the maximum peak temperature shifts to higher values due to the overall exothermic delay. According to Kissinger’s theory, the dipropylene glycol ester reached the polymerization activation energy value of 115 kJ/mol, which is comparable to other low molecular weight itaconic acid derivatives involving a fossil-based epichlorohydrin.^20^ The investigated physical-chemical characterization confirmed DPG-IA’s utility in cured thermosets.

### 3.3 Adhesion Performance

Due to the chemical composition of DPG-IA, the photocurable adhesives are a suitable utility for this synthesized compound. The used itaconic acid is an entirely biobased entering material produced biotechnologically.^16^ Generally, propylene glycol is produced from propylene oxide and water via ring-opening nucleophilic substitution.^23^ In this study, we used a biobased propylene glycol produced from glycerol. Due to the quantitative renewable content, DPG-IA is an appropriate biobased alternative to photocurable petroleum-based adhesives commonly used nowadays.^13^ Generally, adhesive precursors contain multiple functional groups (hydroxyl, amine, carbonyl, and ester) to maximize the intermolecular forces, providing the molecular adhesion toward various adherents.^24^ DPG-IA contains two free hydroxyl groups, promising significant adhesive performance. We performed the adhesion strength measurements investigating the shear adhesion of two different adherents, the acryl abbreviation for poly­(methyl methacrylate) (PMMA) with wood or glass. These two adherents were chosen due to their major hydrophilic character corresponding with a DPG-IA polar molecular structure (see Figure 4). The acryl-wood adhesion of DPG-IA reached 53.6 ± 1.5 kPa of adhesion strength, while the acryl-glass adhesion achieved a value of 83.4 ± 4.4 kPa. We compared the recorded adhesion strength values to the results published in the literature. Liang et al. investigated the adhesive strength of self-healing hydrogel sealant for wound healing, reaching approximately 40 ± 10 kPa adhesive strength.^21^ We also compared our results to the partially biobased polyesters derived from fossil-based aliphatic diols, citric acid, maleic anhydride, and glycidyl methacrylate, achieving an adhesive strength of approximately 86 ± 2 kPa.

**Figure 4:**
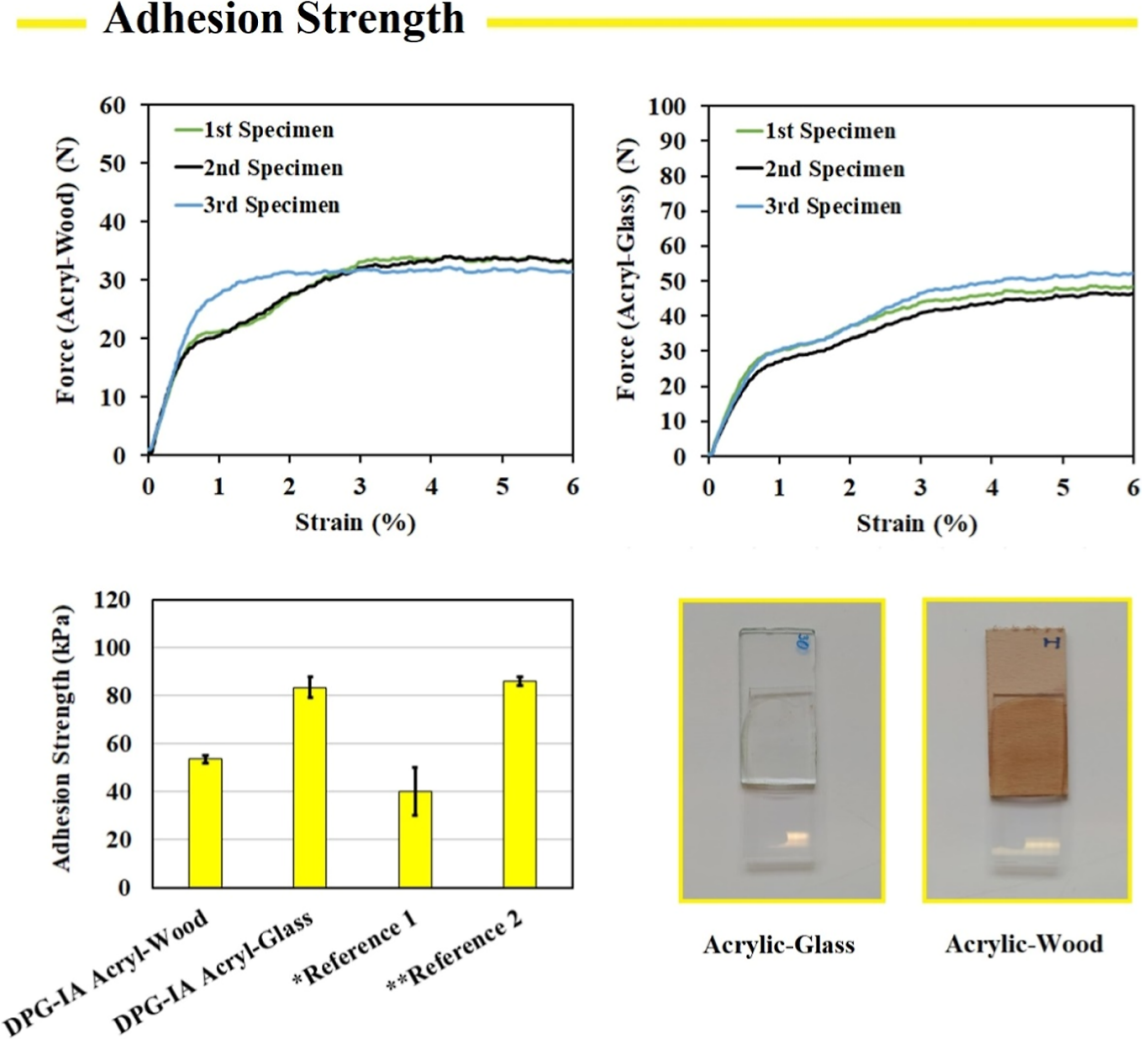
Adhesion strength performance of DPG-IA in acrylic-wood and acrylic-glass adhesives compared to the reference materials from the available literature. *refs 21, **refs 2 and 22

## 4 Conclusion

In summary, the synthesized dipropylene glycol itaconate (DPG-IA) is entirely based on renewable sources. The Fischer esterification leading to the product was performed without a supportive diluent for azeotrope distillation, which increases the scalability and sustainability of the process. The synthesis resulted in a more than 98% yield of the product, structurally verified by FT-IR and volumetric analyses (the acid number and hydroxyl value). The produced reactive ester exhibited a viscosity of 1050 mPa·s at 25 °C with Newtonian behavior. The calculated flow activation energy reached 70.1 kJ/mol. The curing investigation confirmed DPG-IA’s reactivity. According to Kissinger’s theory, the calculated polymerization activation energy reached 115 kJ/mol. The synthesized DPG-IA was applied as a photocurable adhesive on the acryl-wood and acryl-glass adherents. The achieved adhesion strengths reached 53.6 ± 1.5 kPa for the acryl-wood system and 83.4 ± 4.4 kPa for the acryl-glass system. Based on the results, the innovative itaconic acid ester is an appropriate, entirely biobased alternative to the fossil-based photocurable adhesives.

## Supplementary Material



## Notes

Complete measured DSC data (PDF)
