# Supplementary material for: Fully Biobased Thermoset Adhesive Precursor from Itaconic Acid and Propylene Glycol
Source: ACS Omega. 2025 Dec 17;10(51):63565–72. doi: 10.1021/acsomega.5c10583 (PMC12756758; doi:10.1021/acsomega.5c10583)
Supplement: Supplementary file 1 [file ao5c10583_si_001.pdf]

Supporting Information

Fully Bio-Based Thermoset Adhesive Precursor from Itaconic Acid and Propylene Glycol

Vojtěch Jašek[a*], Eliška Kameníková[b] Kamil Novotný[c], Radek Přikryl[a] and Silvestr Figalla[a]

a Institute of Materials Chemistry, Faculty of Chemistry, Brno University of Technology, 61200 Brno, Czech Republic.

b Materials Research Centre, Faculty of Chemistry, Brno University of Technology, 612 00 Brno, Czech Republic.

c Chair of Materials Science and Testing of Polymers, Montanuniversität Leoben, Franz Josef-Straße 18, 8700 Leoben, Austria

*corresponding author: xcjasekv@vutbr.cz

Contents

1. **Complete DSC Results**
    - **Figures S1.** The measured DSC curves investigating the curing characteristics of DPG-IA.

1. Complete DSC Results

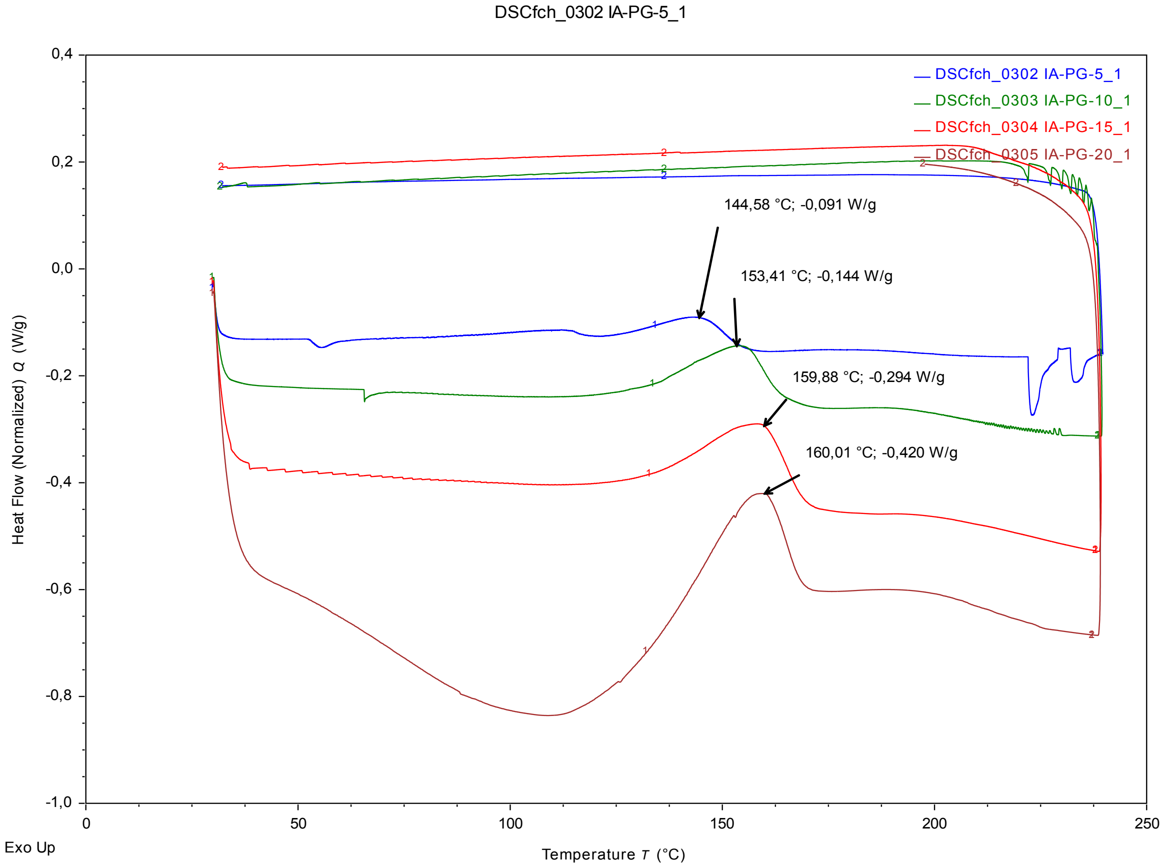

Figure S1. The measured DSC curves investigating the curing characteristics of DPG-IA.
